# Mode-Locked Operation of High-Order Transverse Modes in a Vertical-External-Cavity Surface-Emitting Laser

**DOI:** 10.3390/s24092839

**Published:** 2024-04-29

**Authors:** Tao Wang, Yunjie Liu, Renjiang Zhu, Lidan Jiang, Huanyu Lu, Yanrong Song, Peng Zhang

**Affiliations:** 1 College of Physics and Electronic Engineering, Chongqing Normal University, Chongqing 401331, China; wangt@cqnu.edu.cn (T.W.); 20131121@cqnu.edu.cn (R.Z.); jldan@cqnu.edu.cn (L.J.); 2 National Center for Applied Mathematics in Chongqing, Chongqing Normal University, Chongqing 401331, China; ajk@cqnu.edu.cn; 3 State Key Laboratory of Luminescence and Applications, Changchun Institute of Optics, Fine Mechanics and Physics, Chinese Academy of Sciences, Changchun 130033, China; luhuanyu@ciomp.ac.cn; 4 College of Applied Sciences, Beijing University of Technology, Beijing 100124, China; yrsong@bjut.edu.cn

**Keywords:** mode-locking, vertical-external-cavity surface-emitting laser, high-order transverse mode

## Abstract

Understanding the mechanism of mode-locking in a laser with high-order transverse mode is important for achieving an ultrashort pulses train under more complicated conditions. So far, mode-locking with high-order transverse mode has not been reported in other lasers except the multimode fiber laser. This paper demonstrates robust mode-locking with high-order transverse mode in a Kerr-lens mode-locked vertical-external-cavity surface-emitting laser for the first time, to the best of our knowledge. While the longitudinal modes are locked, continuous mode-locking accompanied by high-order transverse mode up to TEM_40_ is observed. The threshold of the mode-locking is only a little bigger than that of the lasing. After the laser oscillation is built up, the mode-locked pulse train can be obtained almost immediately and maintained until the thermal rollover of the laser. Output powers of 717 mW under fundamental mode and 666 mW under high-order transverse mode are achieved with a 4.3 ps pulse duration and 1.1 GHz pulses repetition rate, and some phenomenological explanations to the related characteristics of the mode-locked operation of high-order transverse mode in the vertical-external-cavity surface-emitting laser are proposed.

## 1. Introduction

Ultra-short pulses with a picosecond to femtosecond duration generated by a mode-locked laser have been widely used in ultrafast time-resolved dynamics, an optical frequency comb, intense field physics, laser medicine, laser micro-processing, and other fields [1,2]. In addition to the well-known dye lasers and solid-state lasers (especially Ti: sapphire lasers) [3,4], mode-locking has also achieved many remarkable results in semiconductor lasers [5,6].

The optically pumped vertical-external-cavity surface-emitting laser (VECSEL), alternatively named as a semiconductor disk laser, follows the geometries of solid-state disk lasers and possesses the advantages of semiconductor lasers. It can produce good beam quality and high output power simultaneously. Meanwhile, the oscillating wavelength of a VECSEL can be tailored using energy gap engineering according to the practical demands, and wavelength tuning, single-frequency operating, and mode-locking can also be accessed conveniently [7,8,9].

In view of the time characteristics of semiconductor carriers, it is generally recognized that a relatively easy method to implement mode-locking in a VECSEL is to use a semiconductor saturable absorption mirror (SESAM) made of the same material system, so to achieve passive mode-locking. Performances of mode-locked VECSEL have been greatly improved since the first passively mode-locked VECSEL was reported [10,11,12,13]. The maximum average output power has been increased to 6.4 W [14], the minimum pulse width has reached 60 fs [15], and the repetition frequency directly arisen from the oscillator has also been pushed to 101.2 GHz [16]. At the same time, various research groups have conducted excellent research on the mechanism of passive mode-locking in a VECSEL, including the effects of dispersion [17], nonequilibrium carrier dynamics, and so on [18].

Interestingly, researchers found another mode-locking method in VECSELs, namely Kerr-lens mode-locking (KLML) or self-mode-locking [19,20]. Some results of KLML need to insert a slit or another Kerr medium in the laser resonator [21,22], while other reports of KLML do not need to place any additional elements in the cavity [23]. This so-called KLML method was controversial at the beginning of its discovery [24]. After the discovery, the explanation of the mechanism of KLML was not consistent. Some people thought that quantum wells at the end position of the semiconductor gain chip are not pumped, which provides saturable absorption to the laser wavelength and acts as an SESAM [25]. At the same time, another viewpoint is that the Kerr effect in the semiconductor gain medium initiates the mode-locking [26]. Currently, it is accepted more widely that the KLML phenomenon in a VECSEL originates from the Kerr effect of the semiconductor gain medium [27].

KLML in solid-state lasers requires strict oscillation conditions, including that the transverse mode of the laser must be a single transverse mode, that is, the fundamental mode. In this case, mode-locking does not involve high-order transverse mode. In fact, the understanding of the mode-locking mechanism has been limited to the time domain [28,29]. Until recently, the spatiotemporal mode-locking (STML) in multimode fibers has been revealed and deeply explored, which extends people’s comprehension of mode-locking to a higher spatial dimension [30,31].

In the KLML of VECSELs, it has been found that mode-locking was assisted by higher-order transverse mode [32]. Based on the beat frequency in the RF spectrum of mode-locking, and further supplemented by the same threshold value of mode-locking and higher-order transverse modes, the authors stated that the emergence of higher-order mode promoted the establishment of mode-locking. However, there was no direct evidence of mode-locking with high-order transverse mode in the above paper.

This work reports a stable Kerr-lens mode-locked VECSEL with high-order transverse mode for the first time, to the best of our knowledge. The evolution of the laser from continuous-wave (CW) operation to the establishment of irregular pulses, and then to CW mode-locking, is observed. Stable CW mode-locked pulses output is realized under different spatial dimensions (i.e., different high-order transverse mode), and the transverse mode order of stable mode-locking can be as high as TEM_40_. The pulse width, pulse repetition rate, and other characteristics of a mode-locked laser are discovered, and the output powers of mode-locked pulses with TEM_00_ and TEM_20_ modes are measured. A preliminary view of the mechanism of the Kerr-lens mode-locked VECSEL with high-order transverse mode is also put forward.

The laser described in this work needs no additional element to start the mode-locking and can be mode-locked in more complicated conditions (i.e., with high-order transverse mode). In other words, it is simpler, more compact, and more robust than other types of mode-locked lasers. Especially, its emitting wavelength is at a 980 nm waveband, which can produce bule ultrashort pulses that train conveniently through second harmonic generation by inserting a nonlinear crystal in the laser cavity, and has unique underwater applications such as optical sensing, high-resolution imaging, high-speed real-time communication, and so on.

## 2. Experimental Results and Discussions

**Figure 1:**
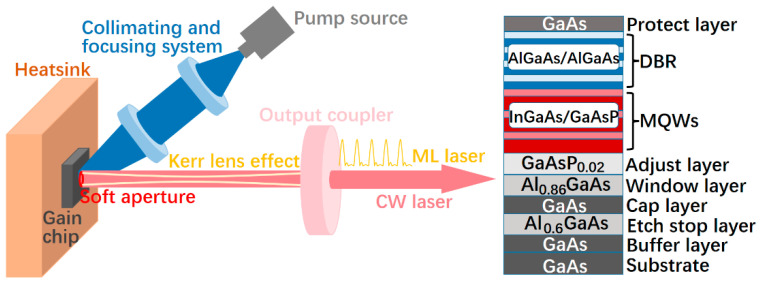
Schematics of the Kerr-lens mode-locked VECSEL (**left**) and the epitaxy structure of the employed gain chip (**right**).

The gain chip used in the experiment is epitaxially grown in reverse sequences, as shown in Figure 1 (right). Firstly, a GaAs buffer layer and an etch stop layer of AlGaAs with high Al composition are deposited on a GaAs substrate, and then a cap layer of GaAs is grown. An AlGaAs window layer with a high barrier to prevent the carriers from surface recombination comes next, and the following is the active region consisting of twelve InGaAs/GaAsP multiple quantum wells (MQWs), which is designed to meet the target laser wavelength of 980 nm. Above the active region is the distributed Bragg reflector (DBR), composed of 30 pairs of alternate AlGaAs layers with high and low Al composition, and the designed center wavelength and high-reflectivity bandwidth of the DBR are 980 nm and 100 nm, respectively. The entire epitaxial wafer is ended by an antioxidant protect GaAs layer.

When the grown wafer is split into small chips with a 4 mm × 4 mm dimension, the epitaxial end face of the chip is metalized with titanium–platinum–aurum sequentially. Then, the chip is bonded to a copper heatsink, and the substrate is removed using chemical etch. An 808 nm fiber-coupled semiconductor laser with a 400 μm core diameter, 0.22 numerical aperture, and 30 W CW output power is used as the pump source, and the pump beam is collimated and then focused onto the gain chip at an angle of about 30°. We use a RoC = −150 mm high-reflectivity coated (for 980 nm wavelength) plane-concave end mirror as the output coupler (OC) to form a straight-line resonator for laser oscillation (see Figure 1 (left)). The total cavity length is about 145 mm, the diameter of the pump spot on the gain chip is about 400 μm, and the size of the beam waist of the fundamental mode of the cavity on the chip is approximately 200 μm. No other intra-cavity elements are employed.

**Figure 2:**
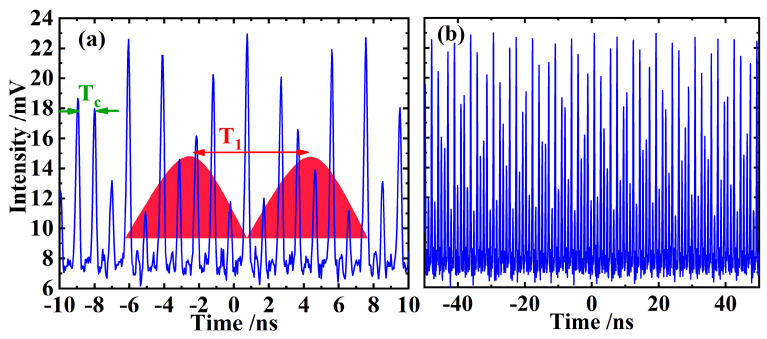
The disordered pulse sequence observed on the oscilloscope (**a**), and the same pulse train on a larger time scale (**b**) when the laser is in pulse operation, but it has not reached a stable mode-locked state yet.

After the laser oscillation is established, it can be changed from continuous wave to pulse operation by properly adjusting the resonator, e.g., finely adjusting OC to change the mode of the laser on the gain chip, or slightly changing the position of the pump spot on the gain chip. However, the generated initial pulses are usually very disordered. The pulse sequence observed on the oscilloscope can be seen in Figure 2a, and the pulse train on a larger scale is shown in Figure 2b, which further illustrates the disorganization of the laser pulses. As shown in Figure 2a, there is a basic period (T_c_) in the pulse sequence, which is exactly equal to the round-trip time of the resonant cavity (formed by OC and the DBR at bottom of the gain chip) used in the experiment.

At the same time, it can also be approximately considered that there is still a modulation period (T_1_) of the pulse amplitude marked by red color block in the pulse sequence. The main reason for drawing the color block in Figure 2a is that there are some pulses whose amplitude changes periodically. Specifically, Figure 2a shows 20 pulses (blue curves), of which the 5th, 7th, 8th, and 10th pulses can be regarded as one group, and the 12th, 14th, 15th, and 17th can be regarded as the other group. The amplitude changes in the two sets of pulses were almost the same. On a longer time scale, the above period does exist, so we draw color blocks to represent its periodicity. The upper edge of the color blocks roughly represents the envelope of the pulse amplitude, and the T_1_ is equal to the distance between the maximum of these color blocks. Although the above envelope modulation in Figure 2a is similar to Q-switched mode-locking, this modulation does not dominate in the pulse train, which is generally chaotic and disorderly.

The above two periods all would be further verified in the later RF measurement. In Figure 2b, the output pulses of the laser are totally disordered, so it cannot be said that the laser is in a mode-locked state but rather in a transition state from CW operation to mode-locking.

**Figure 3:**
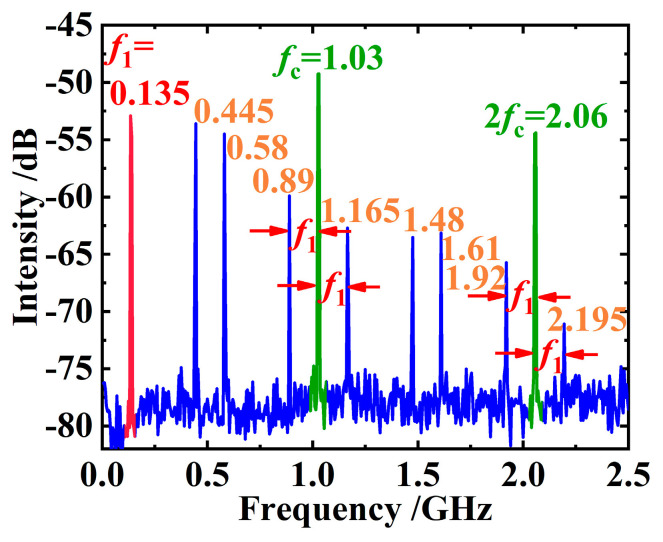
Corresponding RF signals of the disordered laser pulse train produced by a VECSEL that is in pulse operation but not in mode-locking yet. Line *f*_c_ is definitely originated from the cavity round-trip, and line 2*f*_c_ is obviously the second harmonic of *f*_c_. The marked frequency separation is perfectly equal to *f*_1_, and the second, third, seventh, and eighth spectral lines are not so clear.

At this time, the signal appearing on the RF spectrometer is plotted in Figure 3. It can be found that although the spectrum is chaotic in general, some spectral lines still have a clear significance. For example, in Figure 3, the line *f*_c_ = 1.03 GHz corresponds to the pulse fundamental frequency determined by the length of the resonant cavity, that is, to the pulse period T_c_ in Figure 2a. Naturally, the line 2*f*_c_ = 2.06 GHz is the second harmonic of the fundamental frequency of the pulse. As for the first line *f*_1_ = 0.135 GHz in Figure 3, we speculate that it may be caused by a relatively regular modulation embedded into the chaotic pulses, for example, by the pulse amplitude modulation marked by the red color block in Figure 2a, that is, by the period T_1_ in Figure 2a. The lines on the left and right sides of 1.03 GHz in Figure 3 are obviously the difference- and sum-frequency of *f*_c_ and *f*_1_, respectively. Similarly, the lines on the left and right sides of 2.06 GHz are the difference- and sum-frequency signals of 2*f*_c_ and *f*_1_. The meanings of the other spectral lines in Figure 3 are still unclear.

**Figure 4:**
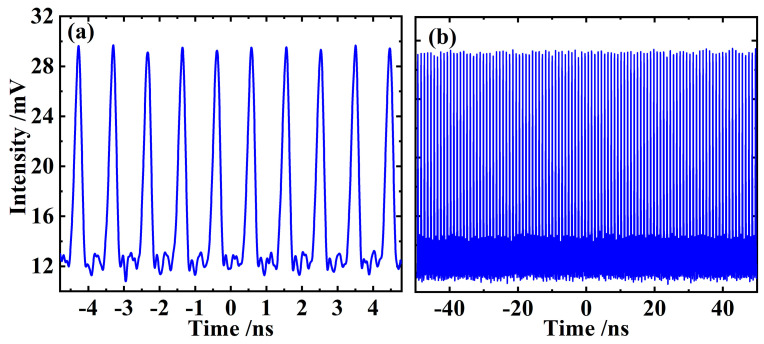
Pulse train of the CW mode-locked VECSEL within 10 ns (**a**), and its extension on a larger time scale of about 100 ns (**b**), which further verifies the good equality and uniformity of the amplitude of pulses.

Further fine tuning of the laser resonator can transform the above disordered pulses into a regular and orderly pulse sequence, i.e., enforce the laser operating at a stable CW mode-locked status, which can be verified from Figure 4. Figure 4a indicates the mode-locked pulse train within 10 ns, where the amplitude of each single pulse is basically the same, and the time interval between pulses is slightly less than 1 ns. Figure 4b shows the pulse train on a larger time scale. Since the amplitude of the pulses shows good equality and uniformity in more than 100 basic cycles shown in the figure, it can be regarded that the laser is in a stable CW mode-locking state.

**Figure 5:**
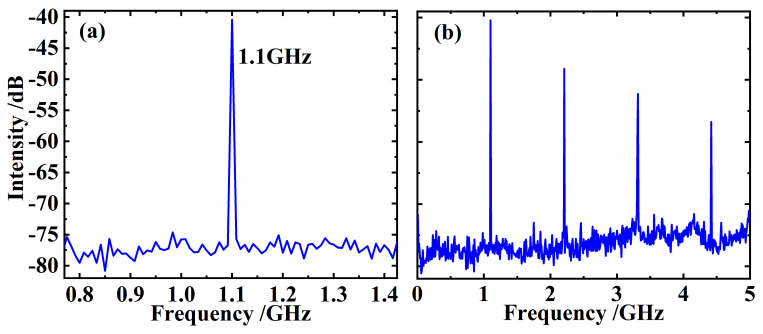
Fundamental RF signal of the CW mode-locked pulses (**a**) showing a 1.1 GHz pulse repetition rate that corresponds to the length of the laser resonant cavity, and some higher-order harmonics of the fundamental signal (**b**).

In order to meet the mode-matching between the laser and the pump spot on the gain chip, so as to reduce the thermal effect and improve the electro-optic efficiency of the laser as much as possible, the length of the laser resonator is chosen to be about 136 mm in this experiment. Figure 5a presents the RF spectrum of the CW mode-locked pulse train, indicating a fundamental pulse repetition rate of 1.1 GHz, strictly corresponding to the length of the resonant cavity used in our experiment. Some higher-order harmonics of the fundamental frequency signal are given in Figure 5b, and its clarity is in distinct contrast to Figure 3.

**Figure 6:**
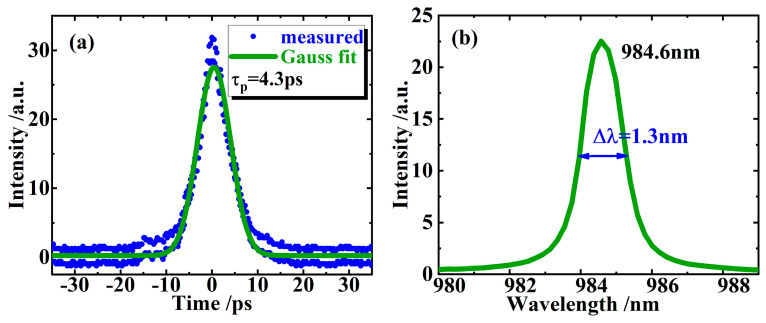
Autocorrelation trace of the CW mode-locked pulses and its Gaussian fitting (**a**). The measured laser spectrum (**b**) indicates an FWHM of about 1.3 nm at 984.6 nm wavelength.

Figure 6a shows the autocorrelation trace of the output pulses, and it can be concluded that the duration of the mode-locked pulse is about 4.3 ps by using Gaussian fitting (a Gaussian fit is chosen here because even if imperfect, it still yields a better performance than a sech fit). Along with the spectral width of 1.3 nm shown in Figure 6b, it can be estimated that the time-bandwidth product of the CW mode-locked pulse is approximately 1.72, almost four times 0.441 (the value of Fourier-transform-limited Gaussian pulse), and this means that a serious frequency chirp is included in the mode-locked laser pulses. Because there are no other dispersion elements in the cavity, we have reason to believe that the nonlinear refractive index caused by the change in the carrier concentration in the active region of the gain chip should be partly or entirely responsible for the chirp included in the mode-locked pulses.

The above CW mode-locking is obtained under the condition of fundamental transverse mode. In view of the absence of any saturable absorption element in the resonator, the mode-locking process is obviously started by the Kerr-lens which resulted from the nonlinear refractive index in the gain chip along with the soft aperture formed by the pump light on the chip (see Figure 1 (left)).

Generally speaking, CW mode-locked solid-state lasers need to work with a single transverse mode, i.e., the fundamental transverse mode or namely the TEM_00_ mode. In contrast, we found in our experiments that a VECSEL can produce stable CW KLML even under high-order transverse mode; that is to say, the transverse modes can be locked simultaneously with the locked longitudinal modes, or spatiotemporal mode-locking can be achieved in a KLML VECSEL.

In the experiment, when the laser is steadily CW mode-locked under a fundamental transverse mode, fine cavity adjustment can make the existing transverse mode deviate from the ideal fundamental mode and maintain the CW mode-locking very well at the same time. It is also possible to carefully tune the resonator under the condition that the CW laser works in multi-transverse mode status, so that the laser can transit from CW operation to stable KLML while the high-order transverse mode is retained.

**Figure 7:**
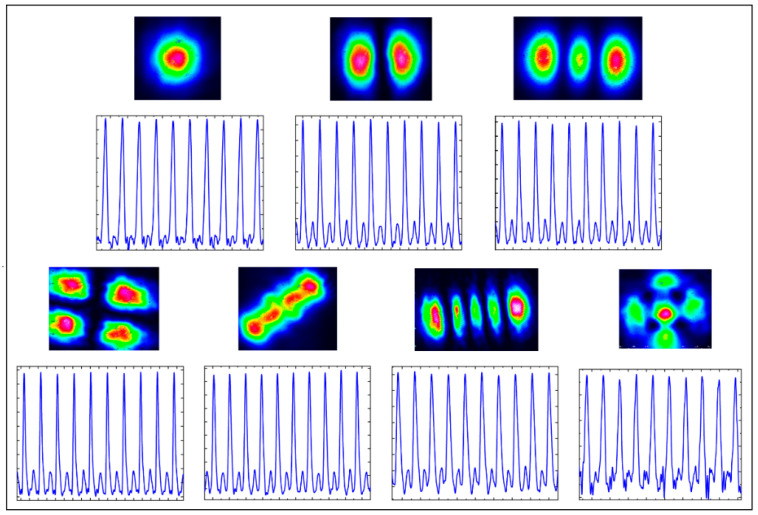
Different transverse modes and their corresponding CW KLML pulse trains. Please note that the differences in the pulse trains are not significant, and the stability and continuity of different pulses trains are all acceptable.

Figure 7 lists different transverse modes that we can obtain stable CW KLML with in the experiments and their corresponding output pulse sequences. We also found that with multi-transverse mode, the CW KLML is even easier to achieve. It has been reported that a higher-order transverse mode may assist the KLML in a VECSEL; however, there is no direct demonstration of CW KLML under higher-order transverse mode in the literature yet.

**Figure 8:**
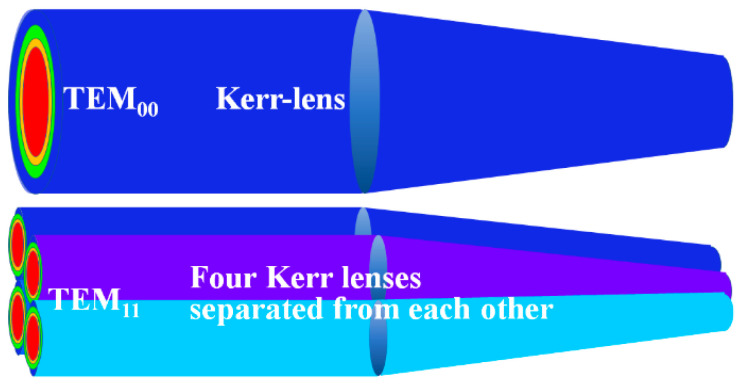
Schematics of the different Kerr-lens of KLML VECSEL with TEM_00_ and TEM_11_ mode. Please note that in the case of TEM_11_ mode, four separated Kerr-lenses working together may make the cavity loss ratio of CW to mode-locked operation greater than that of single Kerr-lens under TEM_00_ mode.

It can be seen from Figure 7 that even under higher-order transverse mode, the status of KLML is still steady enough, and the continuity of the pulses is still satisfactory. It requires further in-depth dynamic analysis to answer why it is difficult to observe CW KLML under high-order transverse mode in a solid-state laser, while stable CW KLML can be established with high-order transverse mode in a VECSEL. We preliminarily suppose that the existence of high-order transverse mode in a solid-state laser means some destructive factors such as polarization degradation and phase detuning, which are unfavorable for the mode-locking of longitudinal modes. By comparison, in a VECSEL, especially a KLML VECSEL supported by the nonlinear refractive index resulting from the change in the carrier density, the presence of high-order transverse mode may represent, to a certain extent, that there may be multiple Kerr-lenses in the laser cavity, as shown in Figure 8.

Without a Kerr-lens, it is obvious that the diffraction loss of the laser cavity under multimode operation is greater than that under single transverse mode. However, if the Kerr-lens works and we consider the cavity loss ratio of the CW operation to mode-locking (*l*_CW_/*l*_ML_), the combined action of the multiple Kerr-lens which arose from the higher-order transverse mode would make the above ratio smaller than that under single-mode. As a result, the KLML process with a higher-order transverse mode can be effectively initiated, even easier than that with the fundamental mode, just as we have observed in the experiment, because in the spatial dimension, the process of mode-locking can be regarded as a spatial self-organization process under the principle of energy optimization.

**Figure 9:**
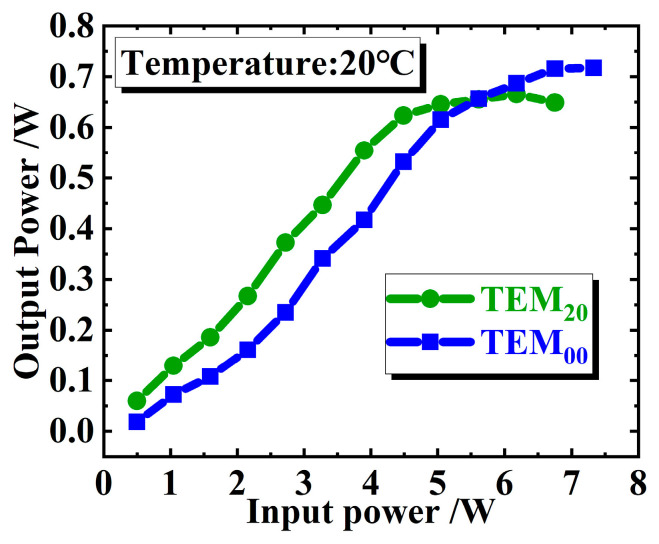
Output powers of the CW mode-locked VECSELs with TEM_00_ and TEM_20_ mode under temperature of 20 °C. When the pump power exceeds 5.6 W, the output power of TEM_20_ begins to decrease and is less than that of TEM_00_.

Obviously, the advantage of a laser with multi-transverse modes is that it can increase the mode volume of the laser in the gain medium and thus increase the output power of the laser. Figure 9 shows the output powers of our CW mode-locked VECSEL with TEM_00_ and TEM_20_ mode, respectively, when the temperature of the heat sink bonded to the gain chip is 20 °C. As can be seen from Figure 9, the output power of the laser with TEM_20_ mode is larger than that of TEM_00_ mode when the incident pump power is below 5.6 W, just as we expected. However, this advantage is not so significant in the experiment. In fact, the maximum output power of the laser with TEM_00_ mode (716 mW) is bigger than the maximum output power of the laser with TEM_20_ mode (666 mW), because the thermal rollover of the laser with TEM_20_ mode occurs earlier. The above phenomenon is understandable, since the diffraction loss of a laser with higher-order transverse mode is relatively larger, and for a laser with small gain, like the VECSEL used in this work, it means lower efficiency and a greater thermal effect; hence, thermal rollover occurs earlier.

It should be noted that no obvious threshold of the mode-locking of high-order transverse mode in a KLML VECSEL was observed in the experiment, that is, the threshold of mode-locking is only a little bigger than that of the CW laser. Once the laser oscillation is built up, the mode-locking can be established almost simultaneously, and the CW mode-locked pulse train can be maintained even beyond the thermal rollover of the laser until the output power drops sharply. We also find that the threshold of the mode-locked laser increases slightly with the increase in the order of the transverse mode, but this increase is not very significant.

## 3. Conclusions

In summary, we have demonstrated robust mode-locking in a KLML VECSEL with high-order transverse mode using a simple straight-line resonant cavity, and stable CW mode-locking accompanied by the transverse mode order up to TEM_40_ is observed. Output powers of 717 mW under fundamental mode and 666 mW under TEM_20_ mode are achieved with the pulse duration of 4.3 ps and a pulses repetition rate of 1.1 GHz. Mode-locking with high-order transverse modes has not been realized in solid-state lasers, nor has it been reported in semiconductor lasers yet. This could inspire research to further explore the mechanism of mode-locking, especially the spatial dynamic process of mode-locking. At the same time, because there is no certain conclusion on the mechanism of mode-locking in a KLML VECSEL yet, the observation of mode-locking with high-order transverse mode in a KLML VECSEL in this paper can also provide a useful reference for understanding the exact physical picture of KLML in a VECSEL. In addition, from the practical point of view, it is expectable that the average output power and the single-pulse energy of a mode-locked laser can be significantly improved by using mode-locking under high-order transverse mode if the thermal effect of the laser can be solved better. Combined with the wide wavelength coverage provided by semiconductor energy band engineering, the advanced mode-locking of high-order transverse mode in a VECSEL would greatly expand the application scenarios of mode-locked semiconductor lasers.

## Data Availability

Data are contained within the article.
